# The Bile Acid Receptor GPBAR-1 (TGR5) Modulates Integrity of Intestinal Barrier and Immune Response to Experimental Colitis

**DOI:** 10.1371/journal.pone.0025637

**Published:** 2011-10-27

**Authors:** Sabrina Cipriani, Andrea Mencarelli, Maria Giovanna Chini, Eleonora Distrutti, Barbara Renga, Giuseppe Bifulco, Franco Baldelli, Annibale Donini, Stefano Fiorucci

**Affiliations:** 1 Dipartimento di Medicina Clinica e Sperimentale, Università degli Studi di Perugia, Perugia, Italy; 2 Dipartimento di Scienze Farmaceutiche, Università degli Studi di Salerno, Salerno, Italy; 3 S.C. di Gastroenterologia ed Epatologia, Azienda Ospedaliera di Perugia, Perugia, Italy; 4 Dipartimento di Medicina e Scienze Biochimiche, Università degli Studi di Perugia, Perugia, Italy; 5 Dipartimento di Scienze Chirurgiche, Radiologiche e Odontostomatologiche, Università degli Studi di Perugia, Perugia, Italy; French National Centre for Scientific Research, France

## Abstract

**Background:**

*GP*-BAR1, a member G protein coupled receptor superfamily, is a cell surface bile acid-activated receptor highly expressed in the ileum and colon. In monocytes, ligation of GP-BAR1 by secondary bile acids results in a cAMP-dependent attenuation of cytokine generation.

**Aims:**

To investigate the role GP-BAR1 in regulating intestinal homeostasis and inflammation-driven immune dysfunction in rodent models of colitis.

**Methods:**

Colitis was induced in wild type and GP-BAR1^−/−^ mice by DSS and TNBS administration. Potential GP-BAR1 agonists were identified by *in silico* screening and computational docking studies.

**Results:**

GP-BAR1^−/−^ mice develop an abnormal morphology of colonic mucous cells and an altered molecular architecture of epithelial tight junctions with increased expression and abnormal subcellular distribution of zonulin 1 resulting in increased intestinal permeability and susceptibility to develop severe colitis in response to DSS at early stage of life. By *in silico* screening and docking studies we identified ciprofloxacin as a GP-BAR1 ligand. In monocytes, ciprofloxacin increases cAMP concentrations and attenuates TNFα release induced by TLR4 ligation in a GP-BAR1 dependent manner. Treating mice rendered colitic by TNBS with ciprofloxacin and oleanolic acid, a well characterized GP-BAR1 ligand, abrogates signs and symptoms of colitis. Colonic expression of GP-BAR1 mRNA increases in rodent models of colitis and tissues from Crohn's disease patients. Flow cytometry analysis demonstrates that ≈90% of CD14+ cells isolated from the lamina propria of TNBS-treated mice stained positively for GP-BAR1.

**Conclusions:**

GP-BAR1 regulates intestinal barrier structure. Its expression increases in rodent models of colitis and Crohn's disease. Ciprofloxacin is a GP-BAR1 ligand.

## Introduction

Bile acids play an essential role in integrating multiple homeostatic functions in the liver and gastrointestinal tract. In recent years these end-product of cholesterol metabolism have been shown to signal through activation of variety of nuclear and cell surface receptors [1]. Activation of Farnesoid-x-receptor (FXR), pregnane-x-receptor (PXR), and constitutive androstane receptor (CAR), along with the vitamin D receptor (VDR), by primary bile acids chenodeoxycholic acid (CDCA) and colic acid (CA) elicits a series of genomic effects that have been deemed essential for regulation of lipid, cholesterol and bile acid homeostasis, local immune response and insulin signalling in intestinal and liver tissues [1], [2]. Knocking down the expression of FXR, the main bile acid receptor, results in a multilevel dysregulation of glucose, lipid, cholesterol and protein metabolism, highlighting the essential role of this receptor in maintaining homeostasis in entero-hepatic tissues [1], [2].

In addition, bile acids exert non-genomic effects [1], [2]. These non-genomic effects have been ascribed to the activation of a cell surface receptor named TGR5 or M-BAR, a member of the rhodopsin-like superfamily of G protein coupled receptor (GPCR), recently christened as a bile acid-activated GPCR (GP-BAR1) [3], [4]. GP-BAR1 is restricted to a limited number of tissues, with the highest expression detected in brown adipose tissue, spleen, macrophages/monocytes, gallbladder and intestine [3]–[5]. In the small and large intestine, GP-BAR1 has been detected in the enteric ganglia of the myenteric and submucosal plexus, in the *muscularis* externa and in the mucosa, in enterocytes of the crypts and villi, while in the cecum and colon the receptor is expressed, thought at lower, in muscle layers and mucosa [6].

In target cells, GP-BAR1 activation by secondary bile acids, lithocolic acid (LCA) and tauro-LCA (TLCA), increases the intracellular concentrations of cyclic adenosine monophosphate (cAMP) and causes the receptor internalization [1]–[4]. In intestinal endocrine L-cells that are higly enriched in receptor expression, GP-BAR1 activation by bile acids and dietary agents stimulates the secretion of glucagon-like peptide (GLP)-1, an insulinotropic hormone that regulates insulin and glucagon secretion along with gastrointestinal motility and appetite [1]–[4], [7].

In addition to its intestinal localization, GP-BAR1 has been detected in peripheral blood derived macrophages and liver macrophages where it exerts an immune-modulatory activity [2], [4]. This activity is inhibitory in nature and manifests itself by attenuation of macrophage's effector functions including reduction of phagocytic activity as well and generation of lipopolysaccharide (LPS)-stimulated cytokines (TNF-α, IL-1α, IL-1β, IL-6, and IL-89 [2], [8].

Despite its role in integrating intestinal homeostasis and glucose metabolism is well defined, it is not known whether GP-BAR1 participates into local regulation of intestinal inflammation and whether its ablation would manifest by an exaggerated inflammatory response to intestinal antigens. Because the expression of GP-BAR1 is highly restricted to the intestine and identification of a regulatory role would be of interest to ground intestine-specific anti-inflammatory therapies, we have investigated whether GP-BAR1 plays a functional role in regulating intestinal homeostasis and inflammation-driven immune response.

## Materials and Methods

C57BL6 were from Harlan Nossan (Udine, Italy) and GP-BAR1 null mice (GP-BAR1-B6 = GP-BAR1^−/−^ mice, generated directly into C57BL/6NCrl background), and congenic littermates on C57BL/6NCrl mice were kindly gifted by Dr. Galya Vassileva (Schering-Plough Research Institute, Kenilworth) [9]. Mice were housed under controlled temperatures (22°C) and photoperiods (12∶12-hour light/dark cycle), allowed unrestricted access to standard mouse chow and tap water and allowed to acclimate to these conditions for at least 5 days before inclusion in an experiment. Protocols were approved by the University of Perugia Animal Care Committee. The ID for this project is #98/2010-B. The authorization was released to Prof. Stefano Fiorucci, as a principal investigator, on May 19, 2010. GLUTag cells were developed originally by Dr. Daniel Druker and were kindly provided by Dr. Fiona Gribble (University Cambridge, UK). Human colon samples were obtained from 6 patients with Crohn's disease (2 female; mean age 43, range 34–61) and 6 subjects (1 female; mean age 51; range 43–64) who underwent colonic resection for colon adenocarcinoma. An informed written consent on the use of biopsies from removed tissues was obtained from each patient. Crohn's disease patients were treated with azathioprine (5 patients) and adalimumab (3 patients) and, in three patients, the reason for surgery was an ileal stricture associated with an abdominal abscess. Control colon samples were obtained from biopsies taken at the intact margins of colonic resections.

### Intestinal permeability studies

Assessment of intestinal permeability towards 4000 Da fluorescent dextran-FITC (DX-4000-FITC) (Sigma-Aldrich, St. Louis, MO) was measured as described by Wang Q., et al. [10]. Briefly, mice were fasted for 6 h and then given DX-4000-FITC by gavage (500 mg/kg body weight, 125 mg (ml). After 4 h, 120 µl of blood was collected from the hearth. The blood was centrifuged at 4°C, 12.000 g for 3 minutes. Plasma was diluted in an equal volume of PBS and analysed for DX-4000-FITC concentration with a fluorescence spectrophotometer (HTS-7000 Plus- plate-reader; Perkin Elmer, Wellesey, MA) at an excitation wavelength of 485 nm and emission wavelength of 535 nm. A standard curve was prepared by diluting FITC-dextrane in non-treated plasma diluted with PBS (1∶3 v/v).

### Animal models of colitis, histopathology analysis and MPO activity

TNBS and DSS colitis were induced as described [11]. Five days after TNBS, 1.5 mg/mouse, or DSS, 5% in drinking water, administration, mice were sacrificed, colons were removed and immediately frozen in liquid nitrogen and stored at −80°C and in formalin. Ciprofloxacin (30 mg/kg i.p.) or oleanolic acid, 0, 1, 1 and 10 mg/kg orally (per os) (both from Sigma-Aldrich, St. Louis, MO) were administered daily for the duration of the experiment. The colon's macroscopic appearance was inspected at the end of the study and graded taking into considering the presence of indurations, edema, thickness and severity of mucosal haemorrhages. Grading was performed in a blinded fashion. For histologic analysis, human and mouse colon samples were fixed in buffered formalin, and routinely prepared 5-µm sections, stained with hematoxylin and eosin (H&E) and mouse colon injury scored as described previously [11], [12].

### Immuno-histochemistry and immuno-fluorescence studies

Human and mouse colon samples were removed and fixed in 10% buffered formalin phosphate, embedded in paraffin and sections (7 µm thickness) processed for immunohistochemistry. Briefly, sections were deparaffinized and, after antigen retrieval, washed in PBS, soaked in 3% H_2_O_2_ for 8 h, and then incubated with 5% bovine serum albumin in PBS with Triton X-100 (0,1%) for 30 min. Sections were then incubated with 10 µg/ml of anti-GP-BAR1 primary antibody (NBP1-39749, Novus Biologicals) or 8 µg/ml rabbit anti-zonulin-1 (Invitrogen), in PBS with 0.3% Triton X-100 and 1% bovine serum albumin, at RT for 2 h. The sections were incubated with biotinylated anti-rabbit IgG 1∶200 (Vector) and then processed by the avidin-biotin-peroxidase method with Vectastain ABC kit (Vector, UK). Diaminobenzidine was used as chromogen. For immunoflurescence, after antigen retrieval by proteinase K, sections were incubated with 8 µg/ml rabbit anti-zonulin-1 in PBS with 0.3% Triton X-100 and 1% bovine serum albumin, at RT for 2 h, and then stained with anti rabbit, phycoerythrin (PE)-conjugated, IgG 1∶200. Cytokine plasma and intestinal levels were measured by commercial ELISA kits (Multi-analyte ELISArray Kit; SAbiosciences A, Qiagen).

### RT-PCRs

Methods for RNA isolation, cDNAs amplification and PCR conditions and analysis have been described previously [13]. All PCR primers were designed with the PRIMER3-NEW software using published sequence data from the NCBI database. Primers were synthesized by MWG BIOTECH. Human (h) and murine (m) sense and antisense primers were as following: mTNFa: (s)acggcatggatctcaaagac and (as) gtgggtgaggagcacgtagt; mIL1b: tcacagcagcacatcaacaa and tgtcctcatcctcgaaggtc; mIL6: ccggagaggagacttcacag and tccacgatttcccagagaac; mIL10: gctggacaacatactgctaacc and ctggggcatcacttctacca; mTGFb1: ttgcttcagctccacagaga and tggttgtagagggcaaggac; mOccludin: cggtacagcagcaatggtaa and ctccccacctgtcgtgtagt; mE-Cadherin: caaagtgacgctgaagtcca and tacacgctgggaaacatgag; mZonulin-1:gggccatctcaactcctgta and agaagggctgacgggtaaat; m18S: accgcagctaggaataatgga and gcctcagttccgaaaacca; mGP-BAR1: ggcctggaactctgttatcg and gtccctcttggctcttcctc; h18S: cggctaccacatccaaggaa and gctggaattaccgcggct; hGPBAR1: cactgttgtccctcctctcc and acactgctttggctgcttg.

### *In silico* and docking studies

Generation of *in silico* model of GP-BAR1 and docking studies of ciprofloxacin and TLCA in the ligand binding site of GP-BAR1 were carried out as described in Materials and Methods S1, accordingly to previously published methods [14]–[21].

### In vitro testing of ciprofloxacin and TLCA on spleen monocytes and GLUTag cells

Mouse monocytes were isolated from spleens of GP-BAR1 wild-type and null mice (C57BL/6BJ6 background) by positive selection using magnetic cell sorting according to the manufacturer's instructions (Miltenyi Biotec) and then cultured in complete RPMI medium as described previously [11], [12]. GLUTag cells were cultured in DMEM with high (4.5 g/litre), glutamine, FBS at 10% and standard P/S antibiotics [22]. GLUTag cells, were treated with increasing concentrations of ciprofloxacin and intracellular cAMP concentration ([cAMP]_i_) evaluated by an EIA kit (Arbor Assays, direct Cyclic AMP assay kit. Spleen monocytes were treated for 30′ with 10 µM ciprofloxacin or TLCA and intracellular cAMP concentration ([cAMP]_i_) evaluated by an EIA kit (Arbor Assays, direct Cyclic AMP assay kit.

To assess anti-inflammatory activities of ciprofloxacin, spleen-derived monocytes from wild type and GP-BAR1^−/−^ were challenged with LPS, 1 µg/ml for 24 hours and TNFα mRNA levels assessed by RT-PCR. RAW 264.7 cells were cultured in DMED with high (4.5 g/litre), glutamine, FBS at 10% and standard P/S antibiotics. RAW cells were treated with increasing concentrations of oleanolic acid [15], [17], and [cAMP]_I_ measured as described above. In addition, RAW cells were challenged with LPS, 1 µg/ml for 24 hours and in the presence of oleanolic acid (1 mM) or TLCA (50 µM) and TNFα released in supernatants assessed by Multi-analyte ELISArray Kit (SAbiosciences A, Qiagen).

### In silico studies and homology modeling

See Materials and Methods S1.

### Statistical Analysis

GraphPad Prism version 3.0 was used for graphics and statistical analyses (GraphPad Software, San Diego, CA, USA). Data are expressed as mean ± SEM. For comparison of more than two groups one-way ANOVA followed by the Tukey test was used. An associated probability<0.05 was considered significant. The Mann-Whitney test was used to compare two groups of data. An associated probability<0.05 was considered significant.

## Results

### GP-BAR1 is expressed in the colon and its expression is modulated by inflammation

**Figure 1:**
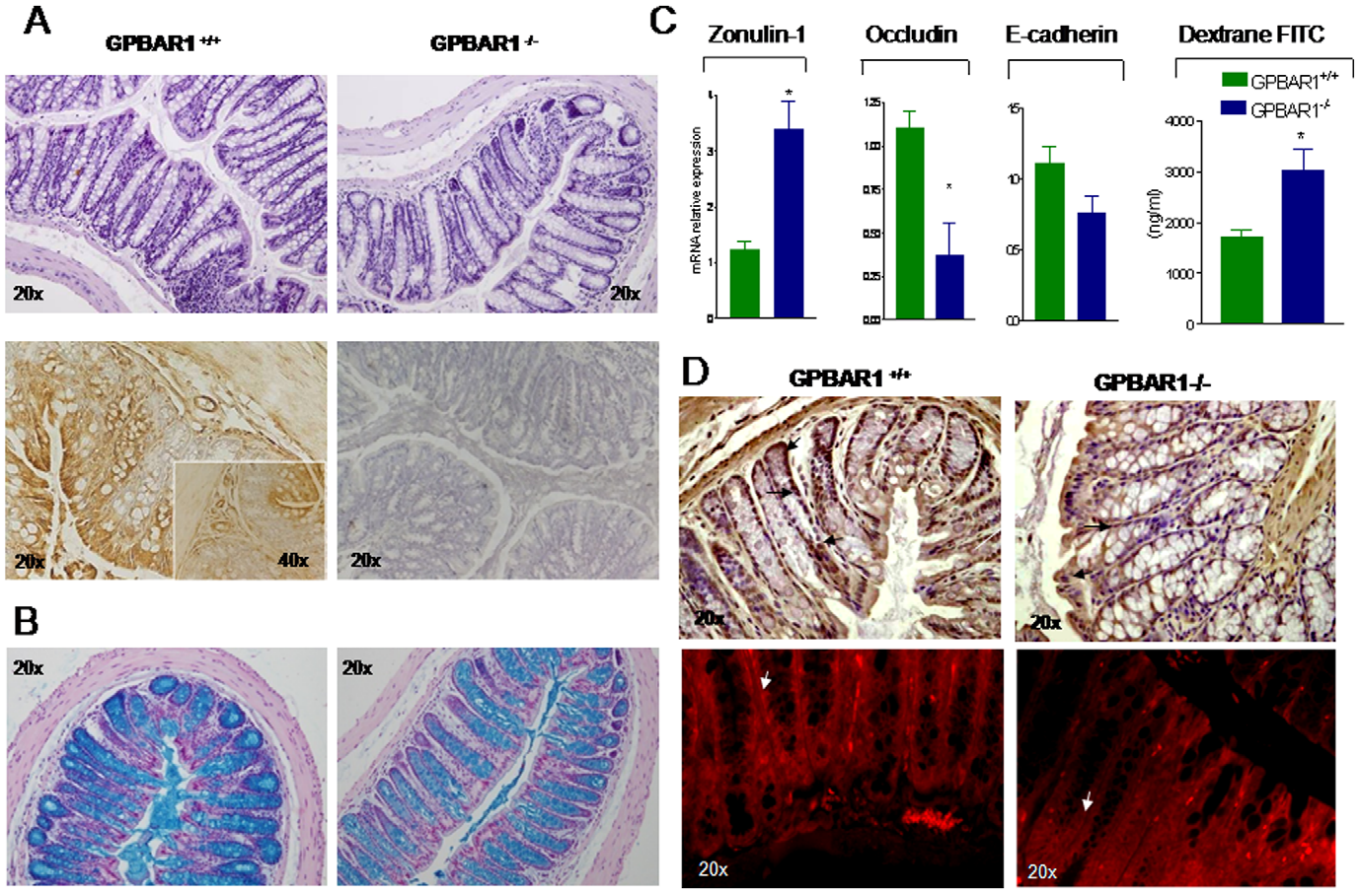
GP-BAR1 gene deletion alters colon structure and function. **Panel A.** Upper panels: H&E staining of colonic sections obtained from 12-month old GP-BAR1^+/+^ and GP-BAR1^−/−^ mice. A significant reduction in colon cellularity and crypt distortion is observed in the colon of GP-BAR1^−/−^ mice. Lower panels: immunohistochemical analysis of GP-BAR1 in wild type mice showing expression in epithelial cells. No staining is observed when the primary antibody is omitted. Original magnification 20× and 40× (insect). **Panel B.** *Alcian blue* staining of colon sections from 12-month old mice showing a severe reduction of mucous cells and their impaired maturation in GP-BAR1^−/−^ mice. Note the marked reduction of *alcian blue* positive cells in the crypts. Original magnification 20×. **Panel C.** RT-PCR analysis of mRNA expression of junctional proteins (zonulin-1, occludin and E-cadherin) in wild type and GP-BAR1^−/−^ mice (n = 6; P<0.05). 12-months GP-BAR1^−/−^ mice also show a significant increase in intestinal permeability to dextrane FITC (n = 6–8; P<0.05 versus naive). **Panel D.** Immuno-histochemical detection and immuno-fluorescence localization of zonulin-1. The staining of the protein is increased in GP-BAR1^−/−^ animals but its cells localization appears to be discontinuous (*arrows*). Original magnification 20×.

To gain insight on the role of GP-BAR1 in regulating intestinal homeostasis, we have first investigated the expression of this receptor in intact colons. Despite the macroscopic inspection of colons obtained from GP-BAR1 mice at the age of 3 (not shown) and 12 months revealed no obvious macroscopic abnormalities, a significant reduction in colon cellularity and crypt distortion was detected at the histopathology analysis of 12-month old GP-BAR1^−/−^ mice (Figure 1A). Analysis of GP-BAR1 expression by immuno-histochemistry (Figure 1A, lower panel) demonstrates a diffuse expression of the receptor in enteric ganglia, *muscolaris* externa and *muscolaris mucosa*, enterocytes and mononuclear cells migrated into the lamina propria. No staining was detected when the primary antibody was omitted (Figure 1A, lower right panel). In addition, GP-BAR1 deficiency results in a marked reduction in the number of *alcian blue* positive mucous cells in the crypt and impairment of their maturation, i.e. a marked reduction along the vertical axis of the crypt (Figure 1B). These changes, as illustrated in Figure 1C and D, correlate with a disarrayed molecular architecture of colonic tight junctions. Indeed by RT-PCR analysis we detected a robust increase in the expression of zonulin-1 mRNA in the colon of GP-BAR1^−/−^ mice, however this increased gene expression associated with an altered sub-cellular distribution of the protein. Thus, while staining for zonulin-1 was uniformly distributed along the apical border of colonocytes and the vascular endothelial cells in wild type mice, a discontinuous pattern was observed in GP-BAR1^−/−^ mice (Figure 1D, arrow). GP-BAR1^−/−^ mice had also a significant reduction in the expression of occluding mRNA (Figure 1C, n = 6; P<0.05), while no changes were detected in the expression of E-cadherin. This dysregulated architecture of tight junctions in GP-BAR1^−/−^ mice associated with a robust increase in intestinal permeability as assessed by measuring dextrane FITC concentrations in the blood (Figure 1C; n = 6–8; P<0.05 versus naive). Phenotypic characterization of mononuclear cells isolated from the *lamina propria* of wild type and GP-BAR1^−/−^ mice revealed no significant changes in total cell numbers nor in the cell phenotype, with the exception of a modest increase in the number of CD8^+^ cells (Figure S1; n = 6; P<0.05).

**Figure 2:**
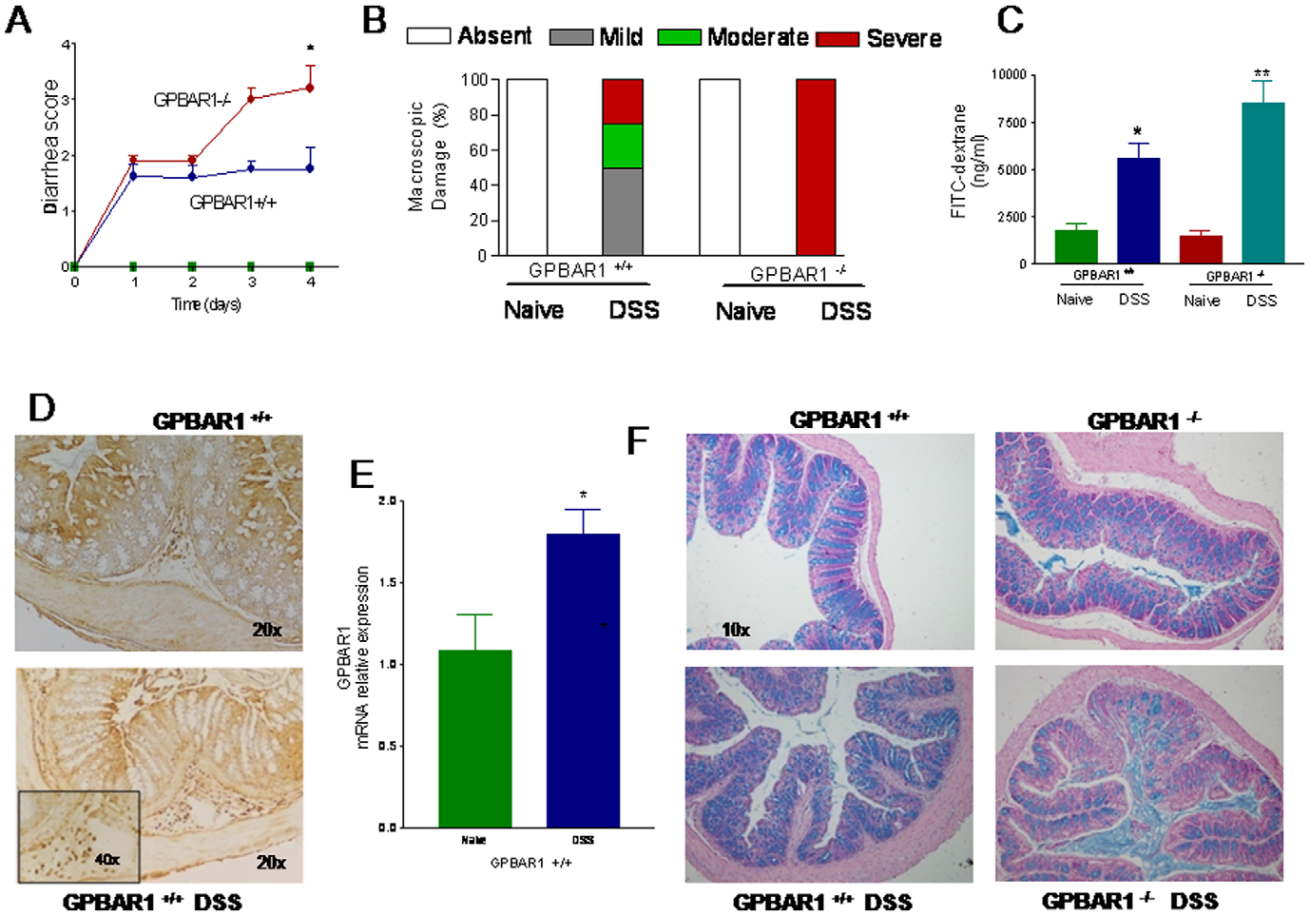
GP-BAR1 gene ablation predisposes to development of severe colitis in response to DSS in 3 months old mice C57Bl/6 mice. **Panel A–C.** GP-BAR1^−/−^ mice develop a severe disease (n = 6; P<0.05), while changes of intestinal permeability were increased in GP-BAR1^−/−^ mice in comparison to wild type mice (n = 10; *P<0.05 versus naive; **P<0.05 versus wild type DSS). **Panel D.** Immuno-histochemical detection of GP-BAR1 expression in the colon of wild type mice challenged with DSS. GP-BAR1 is abundantly expressed in mononuclear cells infiltrating the lamina propria. Original magnification 20× and 40× (inset). **Panel E.** Colon expression of GP-BAR1 mRNA in wild type mice challenged with DSS (n = 6; P<0.05). **Panel F.** Treatment of GP-BAR1^−/−^ mice with DSS results in a dramatic and diffuse loss of *alcian blue*-positive mucous cells. Original magnification 10×.

Because changes in the intestinal permeability might result in an increased tendency to develop injury toward luminal antigens, we have challenged 3-month old wild type and GP-BAR1^−/−^ mice with DSS, a barrier braking agent. The severity of DSS-induced colitis was exacerbated in GP-BAR1^−/−^ resulting in a higher diarrhea and colonic macroscopic injury scores and intestinal permeability (Figure 2A–C). A short course of 5% DSS in drinking water associated with a significant upregulation of colonic expression of GP-BAR1, mRNA and protein (Figure 2D and E), in wild type mice. Increase in GP-BAR1 expression associated with the influx of GP-BAR1 positive mononuclear cells in the colonic lamina propria (Figure 2D, lower panel and inset). Histopathology analysis of alcian blue-stained colons revealed a severe loss of mucous cells in GP-BAR1^−/−^ mice exposed to DSS (Figure 2F). In addition, DSS administration resulted in a robust dysregulation of colon expression of zonulin-1, occludin and E-cadherin mRNAs in both wild type and GP-BAR1^−/−^ mice (Figure S2). Changes in colitis severity were not due different exposure to DSS or reduced daily food and water intakes. Indeed food and water intakes in mice administered DSS were: 4,25±0.3 g/mouse and 6,2±0.5 ml/mouse in wild type mice, and 4.3±0.4 g/mouse and 5,9±0.7 ml/mouse in GP-BAR1^−/−^ mice (none of these values were significant).

### Discovery of ciprofloxacin as GP-BAR1 agonist

**Figure 3:**
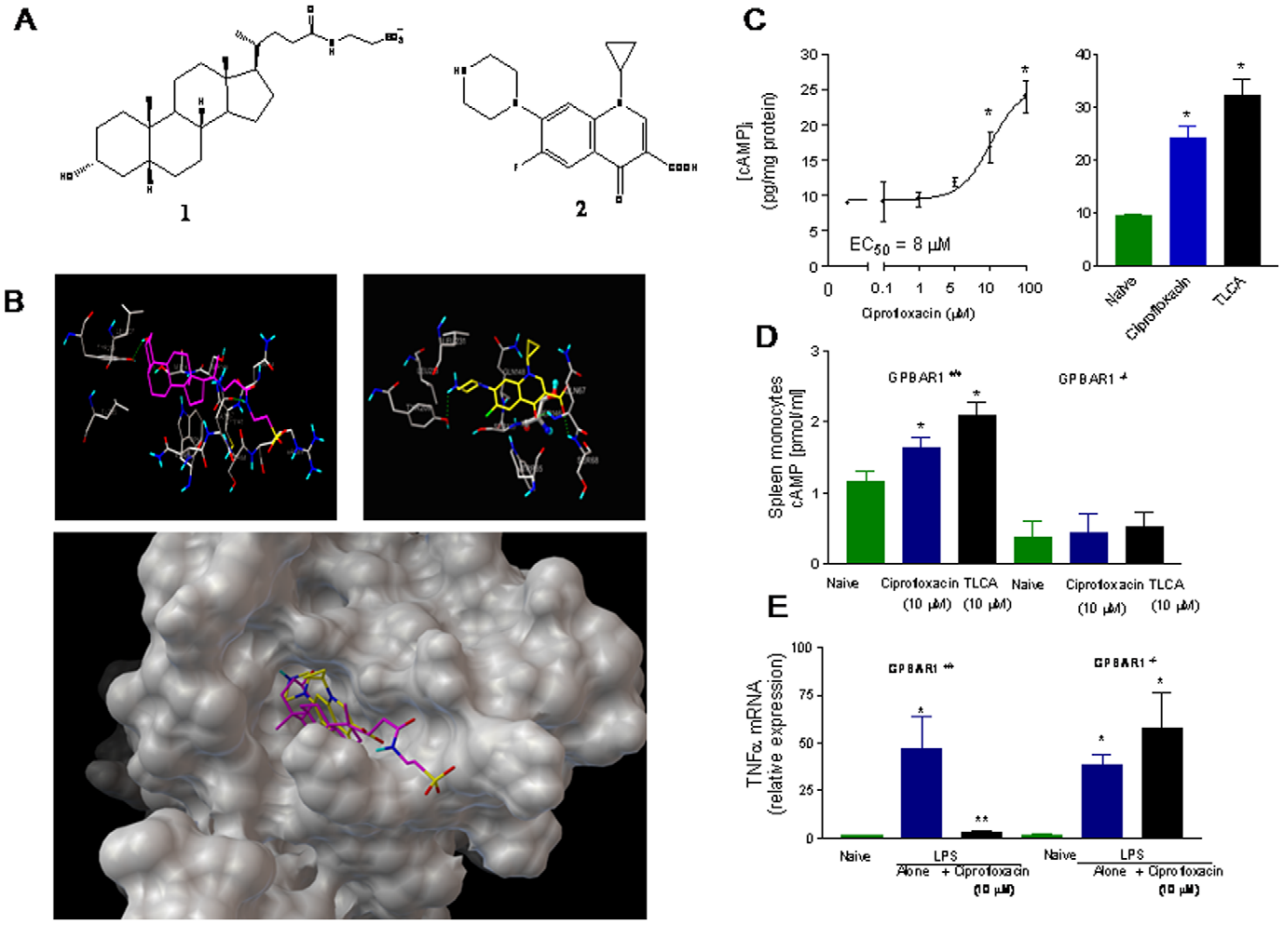
Ciprofloxacin is a GP-BAR1 ligand. **Panel A.** Chemical structure of taurolithocholic acid (1) a natural GP-BAR1 ligand and ciprofloxacin (2). **Panel B.** The predicted GP-BAR1 three dimensional structure was used to analyze the interactions of taurolithocholic acid (lefts) and ciprofloxacin (right) with its binding domain. Both taurolithocholic acid (purple) and ciprofloxacin (yellow) accommodates on the GP-BAR1 binding site (lower panel). **Panel C.** Ciprofloxacin and TLCA increases [cAMP]_i_ in GLUTag cells. N = 4; P<0.05. **Panel D.** Ciprofloxacin and taurolithocholic acid , 10 µM, caused a 2–3 fold increase in [cAMP]i in spleen-derived monocytes isolated from GP-BAR1 wild type mice (n = 4–5; P<0.05), but not in cells isolated from GP-BAR1^−/−^ mice. **Panel E.** Ciprofloxacin, 10 µM, inhibits LPS-induced TNFα release in GP-BAR1^+/+^ monocytes but not in cells isolated from GP-BAR1^−/−^ mice (n = 6; P<0.05 versus naive).

Because only a limited number of GP-BAR1 ligands are available and LCA and TLCA, the two natural ligands, increase intestinal permeability in intact animals and intestinal monolayers [23]–[25], we have carried out an *in silico* screening to identify novel GP-BAR1 agonists. From this *in silico* study we identified ciprofloxacin, a widely used antibiotic [26], as a potential ligand for the receptor (Figure 3A). Because the structure of GP-BAR1 has not yet been solved experimentally, we have carried out an homology modeling study in order to obtain the three dimensional structure of the receptor [14]. For this purposes we have used the human adenosine A2 receptor [14] as template for modeling studies and prediction of GP-BAR1 structure (Figure 3B) and for analysis of the interactions of TLCA and ciprofloxacin with its binding domain (Figure 3B) in order to generate a structure-activity relationship and gain information on their binding mode at atomic level. As shown in Figure 3B, TLCA and ciprofloxacin accommodate on the GP-BAR1 receptor surface interacting with specific aminoacids as further described in Data S1.

Results of docking studies were confirmed by *in vitro* studies in GLUTag cells, a cell line highly enriched in GP-BAR1 [27]. Exposure to ciprofloxacin (Figure 3C) resulted in a concentration-dependent increase of [cAMP]_i_ with an EC_50_ of ≈8 µM (n = 3). Ciprofloxacin, 10 µM, was as effective as 10 µM TLCA (n = 3). To investigate whether these effects were GP-BAR1 dependent, we have then tested the effect of ciprofloxacin on spleen-derived macrophages isolated from wild type and GP-BAR1^−/−^ mice. Figure 3D and E demonstrate that exposure to ciprofloxacin, 10 µM, resulted in a 3-fold increase in [cAMP]_i_ in GP-BAR1^+/+^ (n = 4; p<0.05), but not in GP-BAR1^−/−^ cells. In addition, while ciprofloxacin, 10 µM, completely inhibited TNFα release induced by LPS in GP-BAR1^+/+^ monocytes, this effect was lost in GP-BAR1^−/−^ cells (n = 6; P<0.05 versus naive). Similarly to the effect exerted on cAMP, inhibition exerted by ciprofloxacin on LPS-induced TNFα release was concentration-dependent with an EC_50_ of ≈5 µM (n = 4), data not shown.

### Ciprofloxacin corrects immune dysfunction in TNBS colitis

**Figure 4:**
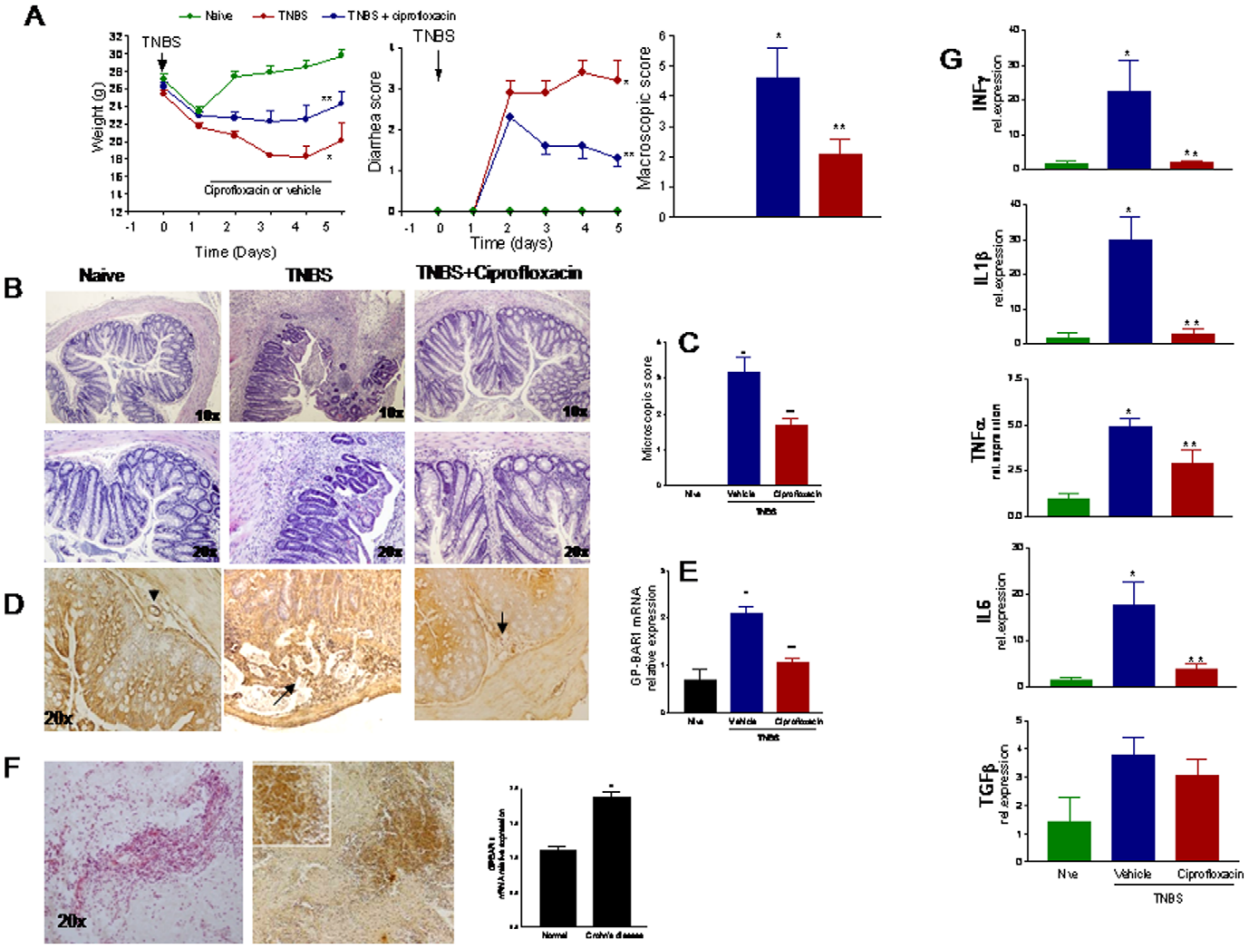
Effects of ciprofloxacin on colitis. **Panel A.** Systemic administration of ciprofloxacin (30 mg/kg/day) protects against development of signs and symptoms of colitis induced by TNBS in GP-BAR1^+/+^ mice (n = 6–8; p<0.05 versus naïve). **Panel B and C.** Ciprofloxacin attenuates histopathology changes induced by TNBS (n = 6–8; p<0.05 versus naïve). Original magnification 10× and 20×. **Panel D and E.** TNBS colitis associates with a robust influx of GP-BAR1 positive cells into the lamina propria of the colon and increased expression of GP-BAR1 mRNA. Mononuclear cells infiltrating the lamina propria show a robust staining for GP-BAR1 (arrow). This upregulation was partially attenuated by ciprofloxacin. **Panel F.** Immuno-hisyochemistry and RT-PCR analysis of GP-BAR1 expression in the colon of Crohn's disease patients. n = 6; P<0.05 versus control subjects. **Panel G.** RT-PCR analysis of expression of signature cytokines in wild type mice exposed to TNBS. Treatment with ciprofloxacin reduces significantly the expression of IL-6, TNF-α, IL-1β and INF-γ mRNAs (n = 6; P<0.05).

Balb/c are highly sensitive to TNBS induced colitis and react to the haptenizing agent by generation of an array of inflammatory mediators with a Th1 signature [11], [12]. As illustrated in Figure 4, systemic administration of ciprofloxacin, 30 mg/kg/day i.p., to TNBS-injected mice protected against development of wasting disease and local signs of inflammation as shown by attenuation of diarrhea score and macroscopic and histopathology scores (Figure 4A–C; n = 6–8; P>0.05 versus TNBS alone). By immuno-histochemistry high levels of expression of GP-BAR1 were detected in immune cells infiltrating the submucosa and mucosa (Figure 4B, arrows). Consistent with these findings, the colonic expression of the GP-BAR1 mRNA increased by 2–3 folds in TNBS treated mice (Figure 4D, E. n = 6 per group, P<0.01 versus naive mice). This pattern of regulation of GP-BAR1 was confirmed by the analysis of the expression of the receptor in colon samples obtained from Crohn's disease patients who underwent right ileo-colon resection. The immune-histochemistry analysis shown in Figure 4F, demonstrates a robust induction of GP-BAR1 expression in areas of colon infiltration by inflammatory cells. The RT-PCR analysis shown in Figure 4F confirmed an increased expression of GP-BAR1 mRNA in the colon of Crohn's disease patients compared to non inflamed colons sampled at the intact margins of resection of colon adenocarcinomas (n = 6; P<0.01).

**Figure 5:**
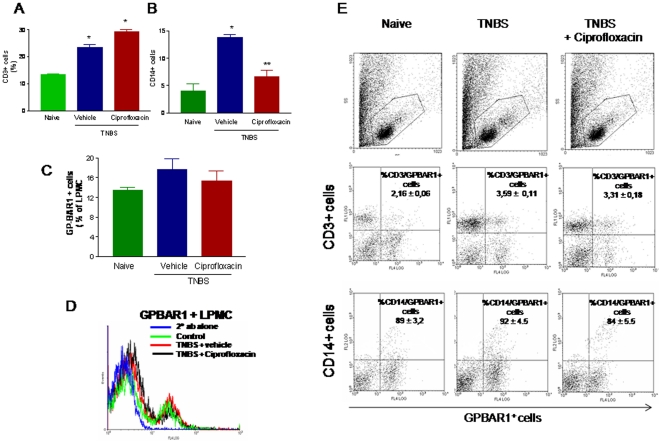
Flow-cytometry analysis of GP-BAR1 expression in unfractioned lamina propria mononuclear cells (LPMC) and LPMC-derived CD3+(A) and CD14+ cells (B) obtained from colons of naïve mice and mice administered TNBS alone and in combination with ciprofloxacin (n = 6; *P<0.05 versus naïve mice, **P<0.05 versus TNBS mice). **Panel C.** Bar graphs of quantitative expression of GP-BAR1 in unfractioned LPMC. **Panel D.** Flow cytometry histogram showing GP-BAR1 protein expression in LPMC in different treatment settings. **Panel E.** Representative Dot plot (forward scatter/ side Scatter) of LMPC obtained from colons of naïve and colitic mice treated with ciprofloxacin. Representative dot plot graphs of relative expression of GP-BAR1 CD3+ and CD14+ cell obtained from LPMC. The numerical values indicate the means ± SE of % double positive cells.

Systemic administration of ciprofloxacin also resulted in a robust attenuation of immune dysfunction caused by TNBS. Thus, while exposure to TNBS caused a 20–30 fold increase in the colonic expression of IL-6, TNFα, IL-1β and IFNγ, these changes were abrogated by co-treating mice with ciprofloxacin (Figure 4G; n = 6–8; p<0.01 versus naive and TNBS). Characterization of LPMC by flow cytometry demonstrates that TNBS administration associates with an increased cellularity mainly due to an influx of CD3^+^ and CD14^+^ cells. The percent of CD3^+^ and CD14^+^ cells increased from 12.1±3.2 and 4.2±1.2 to 23.3±3.1 and 13.8±1.0, respectively (P<0.05 versus naive). While ciprofloxacin, had no effect on the percentage of CD3+ cells, it caused a robust reduction in the number of LP-infiltrating CD14^+^ cells (P<0.01 versus TNBS alone). Characterization of GP-BAR1 expression in these cells revealed that ≈12% of LPMC stained positively for GP-BAR1 in naive mice. This percentage increased in response to TNBS (Figure 5C (A–D). However, while only a small proportion of CD3^+^ cells were co-stained by GP-BAR1 antibody (≈3%), a large proportion of CD14+ cells (≈85%) were GP-BAR1 positive. These relative proportions were not changed by administration of ciprofloxacin (Figure 5E).

### Oleanolic acid, a GP-BAR1 agonist attenuates colitis induced by TNBS

**Figure 6:**
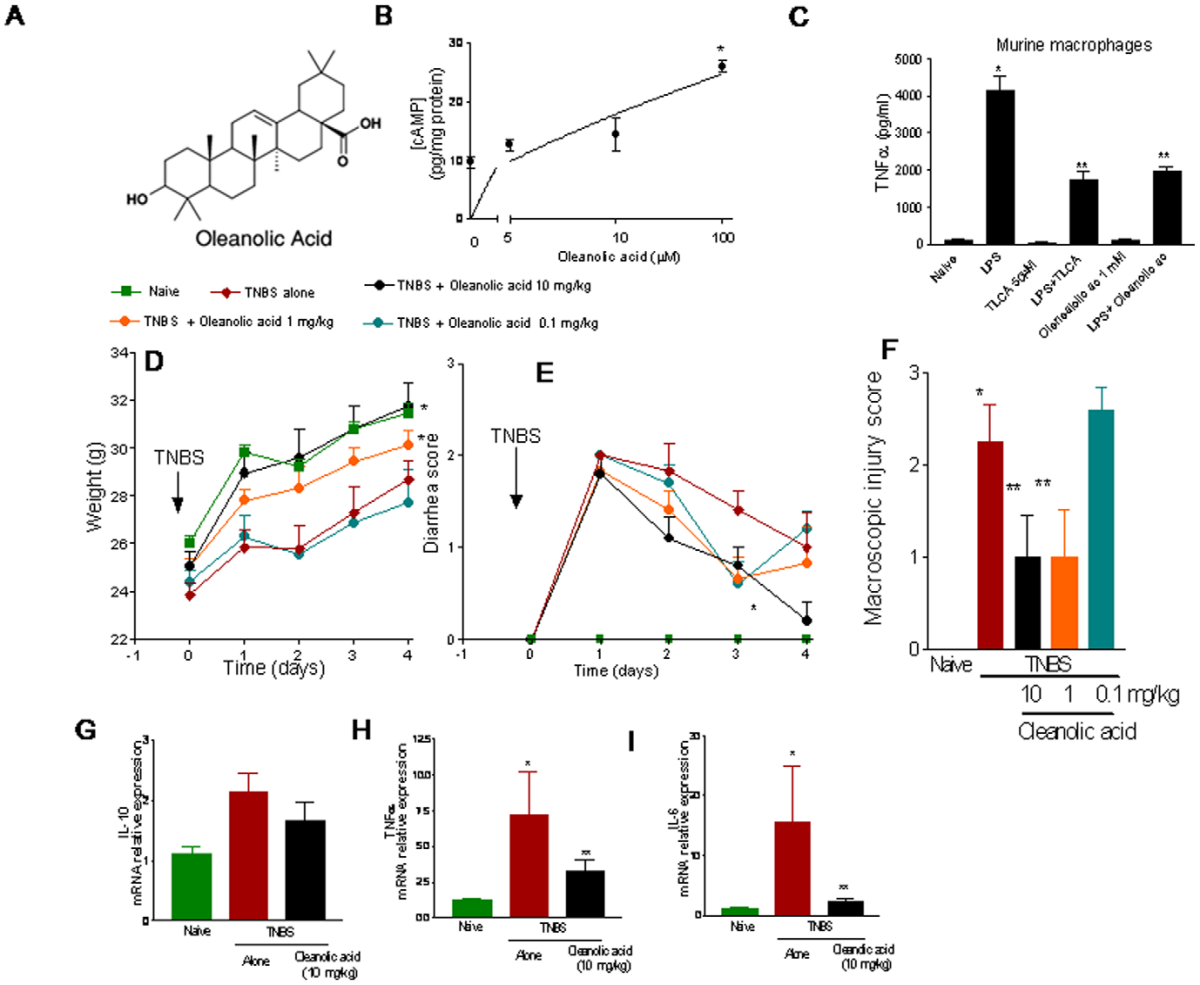
Anti-inflammatory effects of oleanolic acid. Oleanolic acid (**panel A**), a natural GP-BAR1 agonist, exerts anti-inflammatory activity and protects against colitis development in wild type mice. **Panel B.** Oleanolic acid increases [cAMP]_i_ in RAW cells in a concentration-dependent manner. N = 4; *P<0.05 versus basal. **Panel C.** Oleanolic acid reduces TNFα release from murine macrophages exposed to LPS. N = 4. *P<0.05; **P<0.05 versus LPS alone. **Panel D–F.** Oleanolic acid dose-dependently attenuates sign and symptoms of colitis induced by TNBS in wild type mice. N = 5 per group. *P<0.05 versus naive: ** P<0.05 versus TNBS alone. **Panel G–I.** Oleanolic acid treatment leads to a significant reduction in the colonic expression of inflammatory mediators including TNF-α and IL-6. *P<0.05 versus naive: ** P<0.05 versus TNBS alone.

To confirm that GP-BAR1 activation was therapeutically effective in reducing colonic inflammation we have challenged *Balb/c* mice rendered colitic by TNBS treatment with oleanolic acid (Figure 6A), a well established GP-BAR1 ligand [1]–[4], [15], [17]. Activity of oleanolic acid on GP-BAR1 was first investigated by measuring cAMP generation in GLUTag cells. As shown in Figure 6B, oleanolic acid caused a concentration-dependent increase in [cAMP]i and at the concentration of 1 mM, oleanolic acid was effective in reducing TNFα release triggered by LPS from RAW macrophages, confirming that this agent is GP-BAR1 agonist and a TNFα inhibitor (Figure 6C; n = 4; P<0.05). When administered orally at the dose of 0.1, 1 and 10 mg/kg/day for 4 days, oleanolic acid dose-dependently attenuated development of TNBS colitis, as measured by assessing weight loss, diarrhea and macroscopic scores as well as expression of signature cytokines (Il-10, TNFα and IL-6) in the colons (Figure 6D–I; n = 5, P<0.05 versus TNBS alone). Taken together these data demonstrate that GP-BAR1 ligands of chemical or natural origins are effective in reducing inflammation caused by TNBS in wild type mice.

### GP-BAR1 mediates anti-inflammatory activities of ciprofloxacin

**Figure 7:**
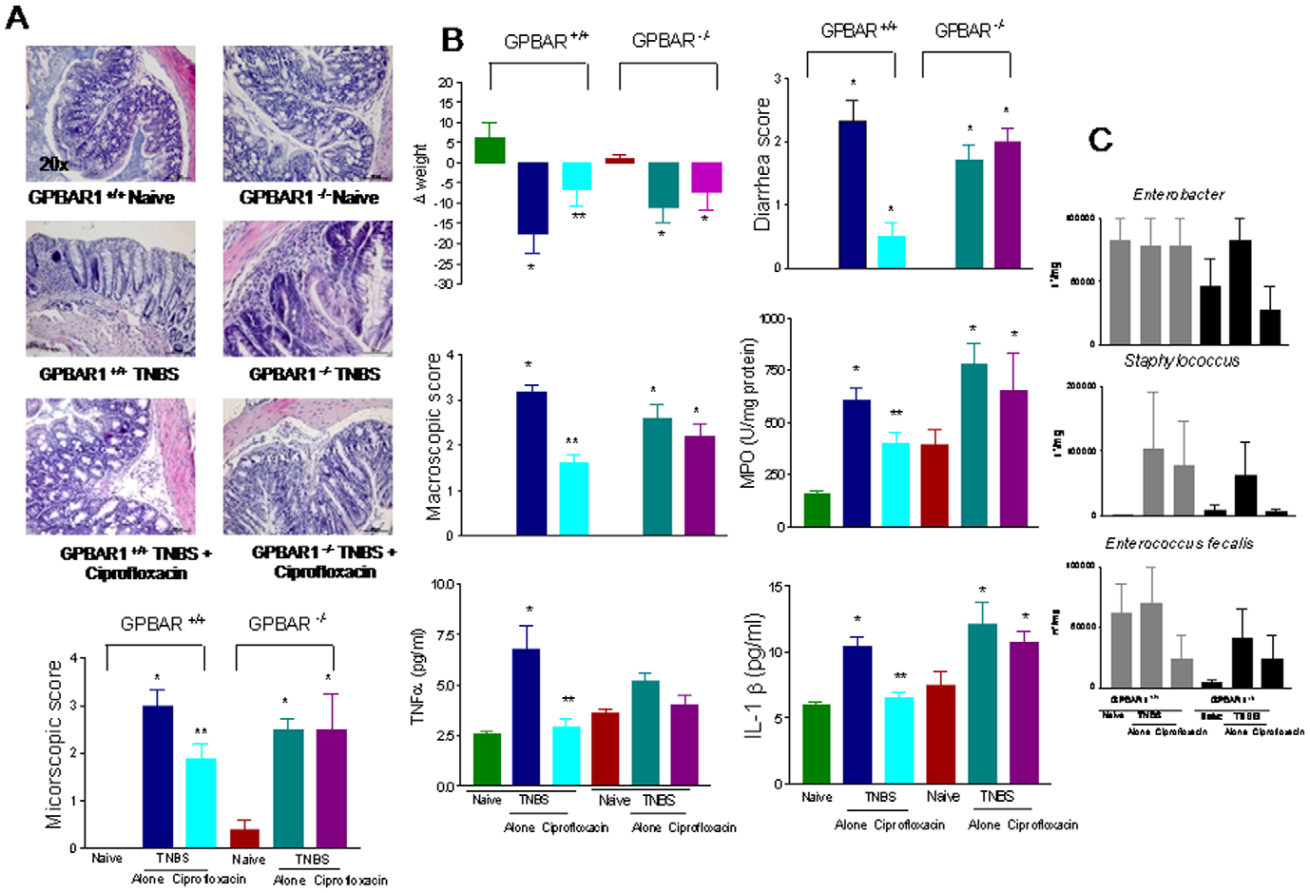
Anti-inflammatory activities of ciprofloxacin are GP-BAR1 dependent. **Panel A.** Ciprofloxacin fails to attenuates colitis induced by TNBS in GP-BAR1^−/−^ mice. Colitis was induced by administering TNBS to GP-BAR1^+/+^ and GP-BAR1^−/−^ mice on a C57BL6J background. Treatment with TNBS results in epithelial degeneration and in intense inflammatory infiltrate. Co- treatment with ciprofloxacin, 30 mg/kg/day, reduces inflammatory infiltrate and epithelial degeneration in wild type mice but not in GP-BAR1^−/−^ mice. **Panel B.** Ciprofloxacin, 30 mg/kg/day, failed to attenuates signs and symptoms of colitis and expression of signature cytokines in GP-BAR1^−/−^ mice challenged with TNBS. (n = 6–8; p>0.05 versus naïve; **P<0.05 versus TNBS). **Panel C.** Systemic administration of ciprofloxacin exerts a comparable antibiotic effect in GP-BAR1^+/+^ and GP-BAR1^−/−^ mice (n = 5 mice per group). Values are not statistically different.

To investigate the role of GP-BAR1 in anti-inflammatory activities of ciprofloxacin, we have then queried whether anti-inflammatory properties of ciprofloxacin would be modulated by the absence of GP-BAR1. Compared to Balb/c mice, administration of wild type and GP-BAR1^−/−^ mice on a C57BL6J background with TNBS and DSS resulted in a mild colitis (Figure 7 (A–C) and Figure S3). TNBS-induced inflammation in wild type mice associated with increased MPO activity, a marker of neutrophils infiltration and increased expression of signature cytokines including TNFα and IL-1β (n = 6–8; p>0.05 versus naive). All these effects were attenuated by ciprofloxacin (30 mg/kg). Administration of TNBS to GP-BAR1^−/−^ mice resulted in a colitis that was partially exacerbated in comparison to that observed in C57BL6J congenic littermates (Figure 7) and that was resistant to treatment with ciprofloxacin, 30 mg/kg/day (n = 6–8; P<0.05 versus naive mice). The lack of efficacy of ciprofloxacin in protecting GP-BAR1^−/−^ mice was independent on its antibiotic activity, because, as shown in Figure 7C, ciprofloxacin exerted a comparable antibiotic activity in both mice strains. Nor different sensitivity to the systemic antibiotic related on a different composition of intestinal flora, because naive wild type and GP-BAR1^−/−^ mice had similar patterns of intestinal bacteria. Finally, the failure of ciprofloxacin to attenuate colitis in GP-BAR1^−/−^ mice was confirmed in a second model of inflammation, i.e. colitis induced by DSS (Figure S3).

## Discussion

GP-BAR1 is G protein coupled receptor activated by secondary bile acids with LCA and TLCA acting *bona fide* as the physiological ligands for the receptor with an EC_50,_ calculated in CHO cells transfected with the human GP-BAR1, of ≈0.5 and ≈0.3 µM, respectively [3], [4]. In the human gastrointestinal tract the GP-BAR1 gene is expressed at the highest levels in the stomach, ileum and colon, but its functional role is largely unexplored. Outside the gastrointestinal tract, the highest level of expression is found in the spleen and resting monocytes. *In vitro* studies have provided compelling evidence that exposure of blood-derived human CD14+ cells to GP-BAR1 agonists results in a negative regulation of macrophage's effector functions and increased [cAMP]_i_ [3], [4], [8], [28]. With this background in mind we have interrogated GP-BAR1 deficient mice to gain insights on the functional role the receptor exerts in regulating intestinal homeostasis. Results from these experiments strongly argue for a mechanistic role for GP-BAR1 in regulating intestinal barrier integrity. Thus, mice lacking GP-BAR1 develop an altered colonic histopatology with a severe alteration in distribution and maturation kinetic of mucous cells as shown by results of alcian blue staining. In addition, GP-BAR1 deficiency results in disruption of molecular architecture of colonic tight junctions that develop with age. Thus, not only the expression of genes encoding for zonulin 1 and occludin was markedly disturbed in GP-BAR1^−/−^ mice, but there was also a significant alteration of subcellular localization of zonulin-1 as demonstrated by immuno-histochemistry. This distortion in the molecular organization of intestinal tight junctions manifests phenotypically with an increased intestinal permeability, that become statistically different from congenic littermates at the age of 12 months. Because alterations in the tight junction architecture is a well recognized hallmark of intestinal inflammation, these data support the notion that GP-BAR1 provides regulatory signals to intestinal epithelial cells.

Little is known on the role of GP-BAR1 on the pathogenesis of human IBDs. However, mapping of locus at chromosome *2q35* i.e. a locus known to be associated with ulcerative colitis (UC) and sclerosing cholangitis (PSC), has revealed an overall association between the *GP-BAR1* single-nucleotide polymorphism, rs11554825, and PSC (odds ratio = 1.1; p = 0.010) and UC (odds ratio = 1.19, p = 8.5×10^−7^), thought that strong linkage disequilibrium precluded further demarcation of GP-BAR1 from neighboring genes [14]. Because rs11554825 polymorphism is a loss-of-function mutation of the GP-BAR1 (TGR5) gene, and an altered intestinal permeability occurs in several genetic abnormalities in IBDs, present results provide a further support to a mechanistic role of GP-BAR1 loss of function mutations in the pathogenesis of IBDs.

While deficiency to maintaining intestinal organization in GP-BAR1^−/−^ manifests with age, GP-BAR1^−/−^ mice were more susceptible than their congenic littermates to develop a colitis when challenged with a barrier braking agent. Thus, at the age of 3 months, when no histopathology abnormalities could be detected, challenging GP-BAR1^−/−^ mice with DSS resulted in a robust exacerbation of local signs of colitis. These changes become apparent after a short course of DSS and were not mirrored by major immunological abnormalities, but a striking increase in intestinal permeability in response to DSS was the major biomarker detected in GP-BAR-1^−/−^ mice in this setting.

An important observation we made in this study was that, not only the absence of GP-BAR1 increases the susceptibility to develop an intestinal inflammation in response to a barrier braking agent, but that the expression of the receptor is robustly modulated by inflammation in rodent models of colitis and Crohn's disease patients. Interestingly the immuno-histochemistry analysis, corroborated by gene expression data, indicates that despite its localization on apical and mucous cells, a significant increase of GP-BAR1 signals in inflamed colons occurs almost exclusively from mononuclear cell infiltrating the colonic lamina propria in mice or from inflammatory cells in Crohn's patients. *Colitides* induced by TNBS and DSS in wild type Balb/c mice associate with a robust influx of mononuclear cells in the lamina propria. A large percentage of these cells stained positively for GP-BAR1 and the phenotypic characterization of LPMC in the TNBS colitis demonstrates that a ≈90% of CD14^+^ cells were GP-BAR1 positive, while only a small proportion of CD3+ cells expressed the receptor strongly indicating a role for GP-BAR1 in regulating cells of innate immunity.

In the search for ligand that could be exploited therapeutically as GP-BAR1 ligand, we have identified ciprofloxacin as a GP-BAR1 agonist. Ciprofloxacin, is widely used in the treatment of infections due to Gram negative bacteria in Crohn's disease. In addition, ciprofloxacin has been shown to increase [cAMP]_i_ in monocytes and macrophages, and by this mean to exert a counter-regulatory effect on cytokine production triggered by LPS [28]. So far the molecular mechanisms mediating these effects were left unknown. By *in silico* screening, docking calculation and *in vitro* experiments we have shown that ciprofloxacin functions as GP-BAR1 agonist. Indeed, not only ciprofloxacin entertains meaningful interaction with key aminoacids in the binding site of GP-BAR1, as demonstrated by docking experiments, but it triggers changes in [cAMP]i in GLUTag cells, an L-like cell line generated from an entero-endocrine tumor and highly enriched in GP-BAR1, and in spleen-derived monocytes [2]–[4], [27]. These effects were lost in GP-BAR1^−/−^ cells, striking indicating that these non-antibiotic effects require the presence of this receptor.

When used to treat colonic inflammation induced by TNBS administration to Balb/c mice, ciprofloxacin effectively protected against development of local signs of colitis and markedly reduced local generation of inflammatory mediators including IL-1β, IL-6, IFNγ and TNFα. Flow cytometry characterization of mononuclear cells infiltrating the lamina propria revealed that, in comparison with animals exposed to TNBS alone, treatment with ciprofloxacin was effective in reducing the number of CD14+ cells from ≈15% to ≈7%. Because expression of GP-BAR1 is detected in ≈90% of CD14+ cells isolated from the lamina propria and ciprofloxacin was administered systemically, these data strongly argue that anti-inflammatory and immuno-regulatory activities of ciprofloxacin are mediated through local and/or systemic activation of GP-BAR1 in CD14+ cells.

While effective at treating abdominal infections in Crohn's disease patients, antibiotics are recognized to carry on a minor role in maintenance therapy in these patients [29]. The lack of efficacy in preventing disease relapse in this setting is poorly explained by pharmacological data. Here, we have made the surprising observation that anti-inflammatory and immune-modulatory actions of ciprofloxacin are lost in GP-BAR1^−/−^ mice. Interrogation of GP-BAR1^−/−^ mice rendered colitic by TNBS or DSS shows that ciprofloxacin looses an important component of its activity when administered to mice that lack this receptor. In both models, ciprofloxacin failed to protect against development of colitis and failed to attenuate colonic influx of neutrophils or expression of signature cytokines such as TNFα and IL-1β in a GP-BAR1-dependent manner. These effects are unrelated to its antibiotic activity, because systemic administration of ciprofloxacin to wild type and GP-BAR1^−/−^ mice caused similar patterns in fecal contents of *Enterobacter*, *Staphilococcus* and *Enteroccocus faecalis* species. Because expression of GP-BAR1 in Crohn's disease might change over time, it could be speculated that analysis of GP-BAR1 expression could help to identify specific subsets of patients that might respond to this agent. Specific clinical data are needed to confirm this hypothesis.

One important observation we made in this study is in addition to ciprofloxacin, oleanolic acid a well characterized GP-BAR1 agonist [15], [17] was also effective in attenuating colitis induced by TNBS. These data strongly argue in favour of a pharmacologically relevant role of GP-BAR1 in modulating colonic inflammation.

In summary, we have shown that GP-BAR1 is involved in regulating intestinal homeostasis and that its absence manifests by an increased intestinal permeability and enhanced susceptibility to develop colitis in response to barrier braking agents. In addition, we have demonstrated that expression of GP-BAR1 increases in response to inflammation in rodent models of colitis and in inflamed tissues obtained from Crohn' disease patients. Finally, we have discovered that ciprofloxacin, a widely used antibiotic, is a GP-BAR1 agonist and that activation of GP-BAR1 with this agent or oleanolic acid, a natural GP-BAR1 ligand, attenuates colon inflammation in rodent models of colitis.

## Supporting Information

Figure S1 **Flow cytometry analysis of lamina propria mononuclear cells isolated from wild type and GP-BAR1^−/−^ mice.** There was no significant difference in the total number of mononuclear cells infiltrating the lamina propria nor in the cells suptypes, with the exception of a in the percentage of CD8+ in GPBAR1^−/−^ mice compared to GPBAR1^+/+^ mice (n = 4; *P<0.05).
(TIF)

Figure S2 **Colon expression of gene encoding for tight junction proteins.** (**A**). Naïve GP-BAR1^−/−^ mice express higher levels of Zonulin 1 mRNA than wild type and exposure to DSS amplify these changes. (**B**). Expression of E-cadherin and occludin mRNA increases in response to DSS, but there is no difference in its up-regulation between wild type and GP-BAR1^−/−^ mice challenged with DSS. N = 6; *P<0.05 versus naive; ** P<0.05 versus DSS.
(TIF)

Figure S3 **Anti-inflammatory activities of ciprofloxacin is lost in GP-BAR1^−/−^ mice challenged with DSS. GP-BAR1−/− mice treated with DSS show an exacerbated colonic inflammation as observed by enhanced loss of body weight, colitis score, macroscopic score (Pane A–C).** DSS treatment also results in a significant increase in colon content of MPO (**Panel D**). All these changes are attenuated by the administration of ciprofloxacin (30 mg/kg) in wild type mice but not GP-BAR1^−/−^ mice. N = 6–8 mice per group. *P<0.05 versus naive.**P<0.05 versus DSS. (**Panel E**) E&E stained colon sections from GP-BAR1−/− mice and wild type mice treated with DSS alone or in combination with ciprofloxacin Magnification 40×. Treatment with DSS results in epithelial degeneration and can be observed an intense inflammatory infiltrate that is enhanced in GP-BAR1^−/−^ mice compared to wild type mice, co- treatment with ciprofloxacin reduces inflammatory infiltrate and epithelial degeneration in wild type mice but not in GP-BAR1^−/−^ mice, as confirmed by microscopic injury score (**Panel F**). DSS treatment increases the colon expression of signature cytokines such as IL-1β and TNFα both in wild type and GP-BAR1−/−. Co-treatment with ciprofloxacin attenuates the expression of these cytokines in wild type but not in GP-BAR1^−/−^ mice (n = 6–8; *p<0.05 versus naïve; **p<0.05 versus wild type DSS treated mice) (**E**).
(TIF)

Materials and Methods S1 **In silico studies and GP-BAR1 homology modeling.**
(DOC)

Data S1 **Results of docking calculation of interaction of ciprofloxacin and TLCA with GP-BAR1 binding site.**
(DOC)
