# Correction: The Bile Acid Receptor GPBAR-1 (TGR5) Modulates Integrity of Intestinal Barrier and Immune Response to Experimental Colitis

**DOI:** 10.1371/annotation/55febddb-0209-4a48-9c14-23df882126a2

**Published:** 2013-01-29

**Authors:** Sabrina Cipriani, Andrea Mencarelli, Maria Giovanna Chini, Eleonora Distrutti, Barbara Renga, Giuseppe Bifulco, Franco Baldelli, Annibale Donini, Stefano Fiorucci

Due to an error, the upper first and third panels in Figure 5E in the article were duplicated. The authors apologize for this error and are making the correct third panel available in a revised figure.

**Figure:**
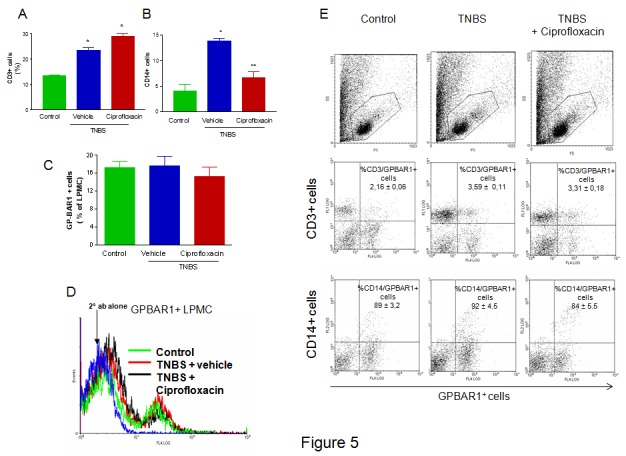


In addition a re-calculation of the data in relation to the GPBAR1 expression in control animals in Figure 5C showed that the basal expression of GP-BAR1 in unfractioned LPMC (Figure 5C) is 17.29 ± 1.36. As a result, the effect on modulation of GPBAR1 expression is not statistically significant.

