# Supplementary figures and images for: The Bile Acid Receptor GPBAR-1 (TGR5) Modulates Integrity of Intestinal Barrier and Immune Response to Experimental Colitis
Source: PLoS One. 2011 Oct 27;6(10):e25637. doi: 10.1371/journal.pone.0025637 (PMC3203117; doi:10.1371/journal.pone.0025637)

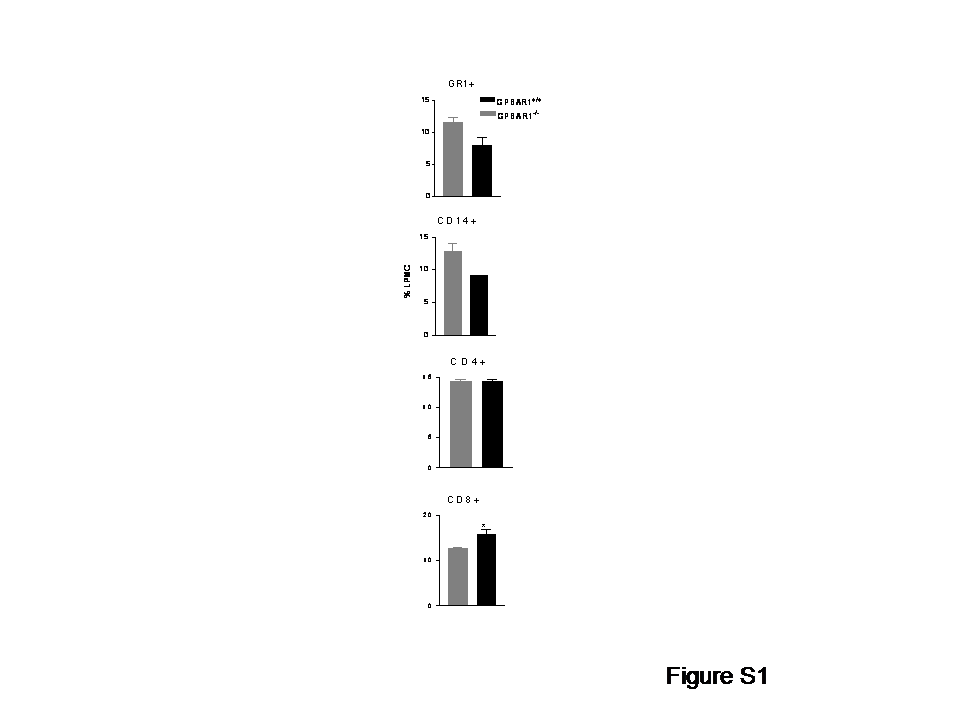

Supplement: Figure S1 — Flow cytometry analysis of lamina propria mononuclear cells isolated from wild type and GP-BAR1−/− mice. There was no significant difference in the total number of mononuclear cells infiltrating the lamina propria nor in the cells suptypes, with the exception of a in the percentage of CD8+ in GPBAR1−/− mice compared to GPBAR1+/+ mice (n = 4; *P<0.05). (TIF) [file pone.0025637.s001.tif]

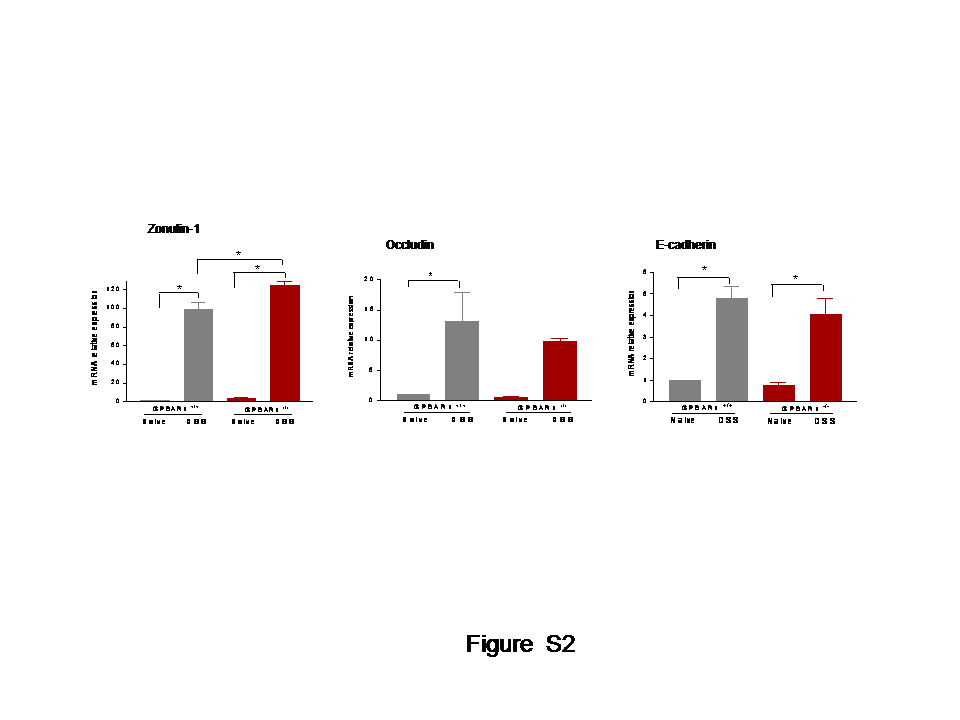

Supplement: Figure S2 — Colon expression of gene encoding for tight junction proteins. (A). Naïve GP-BAR1−/− mice express higher levels of Zonulin 1 mRNA than wild type and exposure to DSS amplify these changes. (B). Expression of E-cadherin and occludin mRNA increases in response to DSS, but there is no difference in its up-regulation between wild type and GP-BAR1−/− mice challenged with DSS. N = 6; *P<0.05 versus naive; ** P<0.05 versus DSS. (TIF) [file pone.0025637.s002.tif]

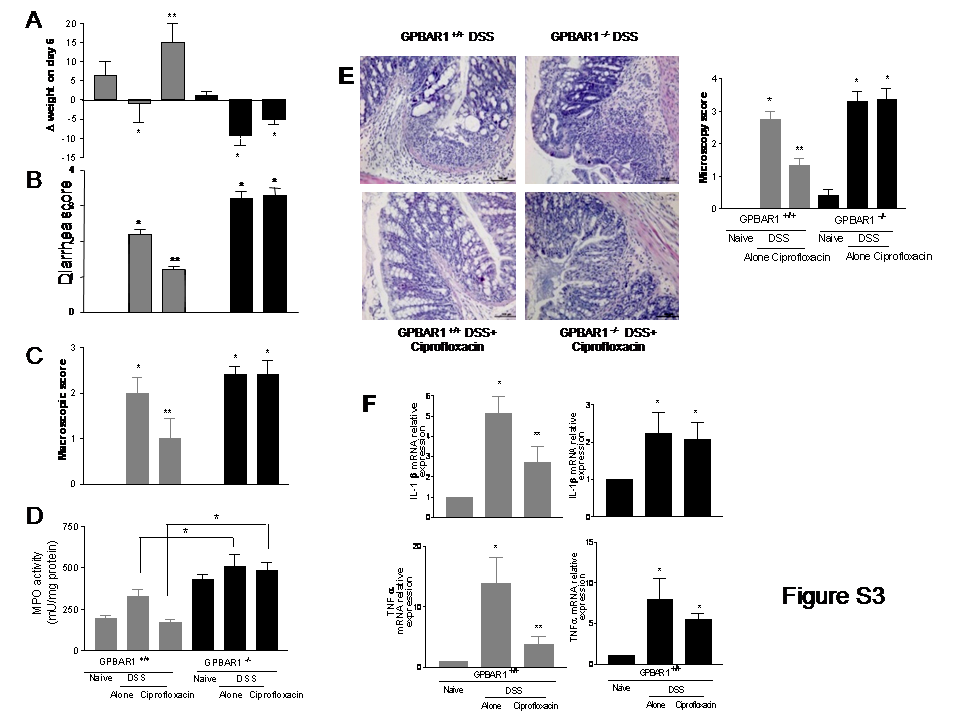

Supplement: Figure S3 — Anti-inflammatory activities of ciprofloxacin is lost in GP-BAR1−/− mice challenged with DSS. GP-BAR1−/− mice treated with DSS show an exacerbated colonic inflammation as observed by enhanced loss of body weight, colitis score, macroscopic score (Pane A–C). DSS treatment also results in a significant increase in colon content of MPO (Panel D). All these changes are attenuated by the administration of ciprofloxacin (30 mg/kg) in wild type mice but not GP-BAR1−/− mice. N = 6–8 mice per group. *P<0.05 versus naive.**P<0.05 versus DSS. (Panel E) E&E stained colon sections from GP-BAR1−/− mice and wild type mice treated with DSS alone or in combination with ciprofloxacin Magnification 40×. Treatment with DSS results in epithelial degeneration and can be observed an intense inflammatory infiltrate that is enhanced in GP-BAR1−/− mice compared to wild type mice, co- treatment with ciprofloxacin reduces inflammatory infiltrate and epithelial degeneration in wild type mice but not in GP-BAR1−/− mice, as confirmed by microscopic injury score (Panel F). DSS treatment increases the colon expression of signature cytokines such as IL-1β and TNFα both in wild type and GP-BAR1−/−. Co-treatment with ciprofloxacin attenuates the expression of these cytokines in wild type but not in GP-BAR1−/− mice (n = 6–8; *p<0.05 versus naïve; **p<0.05 versus wild type DSS treated mice) (E). (TIF) [file pone.0025637.s003.tif]
