# Supplementary material for: The Bile Acid Receptor GPBAR-1 (TGR5) Modulates Integrity of Intestinal Barrier and Immune Response to Experimental Colitis
Source: PLoS One. 2011 Oct 27;6(10):e25637. doi: 10.1371/journal.pone.0025637 (PMC3203117; doi:10.1371/journal.pone.0025637)
Supplement: Materials and Methods S1 — In silico studies and GP-BAR1 homology modeling. (DOC) [file pone.0025637.s004.doc]

**Materials and Methods S1**. **In silico studies and GP-BAR1 homology modeling.**

We focused molecular docking calculations on the agonist binding site at the N-terminal-extracellular portion ^(1)^ portion of the TGR5 receptor.

For the homology modeling studies we referred to the results recently published by Hov et al. ^(1)^ . In their work, the human adenosine A2a receptor (pdb code:3EML) was used as template for the structure studies. In order to obtain the three dimensional model of the GP-BAR1 receptor we used the alignment reported by Hov et al. ^(2)^, see Figure 1.

hTGR5 SPIPKGALGLSLALASLIITANLLLALGIAWDRRLRSPPAGCFFLSLLLAGLLTGLALPT

Template 3EML IMGSSVYITVELAIAVLAILGNVLVCWAVWLNSNLQ-NVTNYFVVSLAAADIAVGVLAIP

hTGR5 LPGLWNQS-RRGYWSCLLVYLAPNFSFLSLLANLLLVHGERYMAVLRPLQ-----PPGSI

Template 3EML FAITISTGFCAACHGCLFIACFVLVLTQSSIFSLLAIAIDRYIAIRIPLRYNGLVTGTRA

hTGR5 RLALLLTWAGPLLFASLPALGWNHWT-------PGANCSSQAIF-PAPYLYLEVYGLLLP

Template 3EML KGIIAICWVLSFAIGLTPMLGWNNCGQSQGCGEGQVACLFEDVVPMNYMVYFNFFACVLV

hTGR5 AVGAAAFLSVRVLATAHRQL----------------------------------------

Template 3EML PLLLMLGVYLRIFLAARRQLNIFEMLRIDEGLRLKIYKDTEGYYTIGIGHLLTKSPSLNA

hTGR5 ------------------------------------------------------------

Template 3EML AKSELDKAIGRNTNGVITKDEAEKLFNQDVDAAVRGILRNAKLKPVYDSLDAVRRAALIN

hTGR5 ------------------------------------------------------------

Template 3EML MVFQMGETGVAGFTNSLRMLQQKRWDEAAVNLAKSRWYNQTPNRAKRVITTFRTGTWDAY

hTGR5 -----LTWRQARAQAGAMLLFGLCWGPYVATLLLSVLAYEQRPPLGPGTLLSLLSLGSAS

Template 3EML RSTLQKEVHAAKSLAIIVGLFALCWLPLHIINCFTFFCPD-CSHAPLWLMYLAIVLSHTN

hTGR5 AAAVPVAMGLGDQRYTAPWRAAAQRCLQGL

Template 3EML SVVNPFIYAYRIREFRQTFRKIIRSHVLRQ

Figure 1. The sequence alignment of 3EML and human TGR5.

The alignment was used as input for the automated homology modeling program MODELER ^(3-6)^ . The number of generated loops was set to 5 along with high optimization level for models and loops. The generated models of GP-BAR1 showing the lowest energy, lowest restraint violations were selected for further refinement of the main and side chains. Hydrogen atoms were added to the selected models using Maestro 8.5 software package ^(7)^ . In details the charges of side chains were assigned considering their pK_a_ at physiological pH. The geometry of the model for the target was optimized in three steps:

1) optimization of model with the added hydrogen atoms by Amber force field (steepest descent method, 500 steps and convergence threshold of 0.05 kJ mol^-1^ Å^-1^). 2) optimization of the side chains using the same criteria of step 1. 3) optimization of the whole structure by Polak-Ribiere Conjugate Gradient (PRCG, 9 x 10^7^ steps, convergence threshold 0.001 kJ mol^-1^ Å^-1^). The quality of the obtained model for GP-BAR1 was validated using the software PROCHECK ^(8)^ .

The homology model for GP-BAR1 (see Figure 2) was used in molecular docking calculations using AutoDock 4.2.


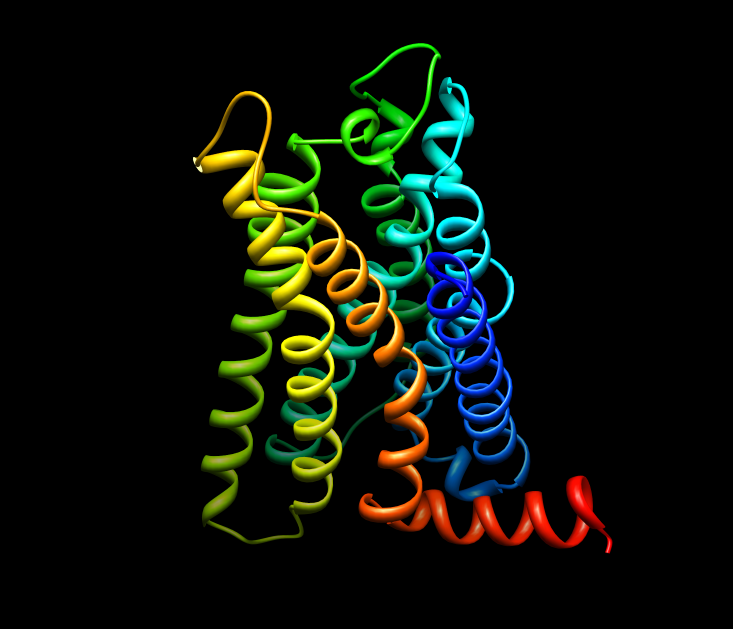


Figure 2. Predicted three dimensional model of the TGR5 receptor.

To achieve a representative conformational space during the docking studies and for taking into account the variable number of active torsions, 10 calculations consisting of 256 runs were performed, obtaining 2560 structures for each ligand. The Lamarckian genetic algorithm was employed for docking calculations, choosing an initial population of 450 randomly placed individuals. The maximum number of energy evaluations and of generations was set up to 5 x 10^6^ and the maximum number of generations to 6 x 10^6^. A mutation rate of 0.02 and a crossover rate of 0.8 were used, and the local search frequency was set up at 0.26. Results differing by less than 2 Å in positional root-mean-square deviation (rmsd) were clustered together and represented by the result with the most favourable free energy of binding. For all the docked structures, all bonds were treated as active torsional bonds except the amide bonds.

**References**

1. Tiwari A, and Maiti P. TGR5: an emerging bile acid G-protein-coupled receptor target for the potential treatment of metabolic disorders. Drug Discovery Today 2009; 14:523-30.
2. Hov JR, Keitel V, Laerdahl JK, et al. [Mutational characterization of the bile acid receptor TGR5 in primary sclerosing cholangitis.](http://www.ncbi.nlm.nih.gov/pubmed/20811628) PLoS One 2010; 5:1-13.
3. Eswar N, Marti-Renom MA, Webb B, et al. Comparative Protein Structure Modeling With MODELLER. Current Protocols in Bioinformatics. John Wiley & Sons, Inc. 2006; Supplement 15, 5.6.1-5.6.30,.
4. Marti-Renom MA, Stuart A, Fiser A , et al. Comparative protein structure modeling of genes and genomes. Annu Rev Biophys Biomol Struct 29, 291:325, 2000
5. Sali A, and Blundell TL. Comparative protein modelling by satisfaction of spatial restraints. J Mol Biol 1993; 234:779-815.
6. Fiser A , Do RK , et al. Modeling of loops in protein structures, Protein Science 2000; 9: 1753-1773.
7. MacroModel, version 8.5, Schrödinger LLC, New York, NY, 2003.
8. Laskowski R A, MacArthur M W, Moss D S,et al. PROCHECK- a program to check the stereochemical quality of protein structures. 1993; *J App Cryst*  26:283-291.
