# Supplementary material for: The Bile Acid Receptor GPBAR-1 (TGR5) Modulates Integrity of Intestinal Barrier and Immune Response to Experimental Colitis
Source: PLoS One. 2011 Oct 27;6(10):e25637. doi: 10.1371/journal.pone.0025637 (PMC3203117; doi:10.1371/journal.pone.0025637)
Supplement: Data S1 — Results of docking calculation of interaction of ciprofloxacin and TLCA with GP-BAR1 binding site. (DOC) [file pone.0025637.s005.doc]

**Data S1.** **Results of docking calculation of interaction of ciprofloxacin and TLCA with GP-BAR1 binding site.**

As reported by Tiwari et al., (1) the agonists bind the N-terminal-extracellular tail of GP-BAR1 we focused our docking calculations around this protein portion. Moreover, as reported by others research groups the hydroxyl group at C-3 of TLCA is involved in hydrogen bond interactions with the receptor, and removal of this group causes decrease in potency toward GP-BAR1 activation (2,3) . As shown in Figure 3B-D, the TLCA accommodates on the GP-BAR1 receptor surface, and it is involved in van der Waals interactions with TRP65, ASN66, SER68, ARG69, ASN144, CYS145, SER146, LEU231, LEU235. The OH at C-3 interacts with hydroxyl groups of TYR209 and SER147, the NH of the taurine interacts with CO of GLN67, also in agreement with the hydrogen bond model proposed by Tiwari et al. and the sulfate group protrudes toward the solvent. Docking studies demonstrate that ciprofloxacin accommodates into the ligand binding site of the GP-BAR1. The CO and COOH groups of the antibiotic generate hydrogen bond interactions with GLN67 and SER68 respectively, and also piperazine group forms an hydrogen bond with the OH of TYR209. Moreover the molecule establishes hydrophobic interactions with the cavity pocket created by SER146, SER147, GLN148, LEU231, LEU213, TRP65, ASN66, indicating that the binding pose of ciprofloxacin in the N-terminal region of GP-BAR1 is the same of TLCA and is compatible with a putative agonist activity on GP-BAR1 .

**References**

1. Tiwari A, and Maiti P. TGR5: an emerging bile acid G-protein-coupled receptor target for the potential treatment of metabolic disorders. Drug Discovery Today 2009; 14:523-30.

Sato H, Macchiarulo A, Thomas C, et al. J Med C hem. 2008, 51, 1831-1841

1. Pellicciari R, Sato H, Gioiello A, et al *J. Med. Chem.* 2007, *50,* 4265-4268
